# The Concentration of Memantine in the Cerebrospinal Fluid of Alzheimer’s Disease Patients and Its Consequence to Oxidative Stress Biomarkers

**DOI:** 10.3389/fphar.2019.00943

**Published:** 2019-08-28

**Authors:** Martin Valis, David Herman, Nela Vanova, Jiri Masopust, Oldrich Vysata, Jakub Hort, Zbysek Pavelek, Blanka Klimova, Kamil Kuca, Jan Misik, Jana Zdarova Karasova

**Affiliations:** ^1^Department of Neurology, Faculty of Medicine and University Hospital Hradec Kralove, Charles University in Prague, Hradec Kralove, Czechia; ^2^Biomedical Research Center, University Hospital Hradec Kralove, Hradec Kralove, Czechia; ^3^Department of Toxicology and Military Pharmacy, Faculty of Military Health Sciences, University of Defense in Brno, Hradec Kralove, Czechia; ^4^Department of Pharmaceutical Chemistry and Pharmaceutical Analysis, Faculty of Pharmacy in Hradec Kralove, Charles University, Czechia; ^5^Department of Psychiatry, Faculty of Medicine and University Hospital Hradec Kralove, Charles University in Prague, Hradec Kralove, Czechia; ^6^Department of Neurology, 2nd Faculty of Medicine, Charles University and Motol University Hospital, Prague, Czechia; ^7^International Clinical Research Center, St. Anne’s University Hospital Brno, Brno, Czechia

**Keywords:** memantine, Alzheimer’s disease, clinical study, cerebrospinal fluid concentrations, oxidative stress, biomarkers

## Abstract

Memantine is a noncompetitive *N*-methyl-d-aspartate (NMDA) receptor antagonist utilized as a palliative cure for Alzheimer’s disease. This is the second study examining the memantine concentrations in cerebrospinal fluid. The previously published study enrolled six patients, and three of them were theoretically in a steady state. In our study, we enrolled 22 patients who regularly used a standard therapeutic dose of memantine (20 mg/day, oral administration) before the sample collection. Patients were divided into four groups, according to the time of plasma and cerebrospinal fluid collection: 6, 12, 18, and 24 h after memantine administration. The cerebrospinal fluid samples were also assessed for selected oxidative stress parameters (malondialdehyde, 3-nitrotyrosine, glutathione, non-protein thiols, and non-protein disulfides). The plasma/cerebrospinal fluid (CSF) ratio for all time intervals were within the range of 45.89% (6 h) to 55.60% (18 h), which corresponds with previously published findings in most patients. The other aim of our study was to deduce whether the achieved “real” memantine concentration in the central compartment was sufficient to block NMDA receptors. The IC_50_ value of memantine as an NMDA antagonist is in micromolar range; the lowest limit is 112 ng/ml (GluN2C), and this value was achieved only in three cases. The memantine cerebrospinal fluid concentration did not reach one quarter of the IC_50_ value in five cases (one patient was excluded for noncompliance); therefore, the potency of memantine as a therapeutic effect in patients may be questionable. However, it appears that memantine therapy positively affected the levels of some oxidative stress parameters, especially non-protein thiols and 3-nitrotyrosine.

## Introduction

Alzheimer’s disease (AD) is a neurodegenerative disease that manifests as a decline in cognitive functions and subsequent behavioral changes in daily activities (Klimova et al., 2016). AD accounts for 60–70% of all dementia cases. With an increase in the aging population, the incidence of AD is expected to rise, especially in developed countries. Thus, to reduce the burden on patients and their caregivers, and the economic and social impacts for society, efficient pharmacological and non-pharmacological therapies must be employed (Klimova et al., 2017). In this study, we focused on one of the rare effective pharmacological therapies, memantine, a noncompetitive *N*-methyl-d-aspartate (NMDA) receptor antagonist.

AD is associated with multiple etiologies and oxidative. Nitrosative stress appears to be an important contributing factor to disease pathogenesis (Huang et al., 2016). The accumulation of amyloid beta peptide (Aβ) in senile plaques (Zhao and Zhao, 2013), in the presence of redox-active metal ions, may lead to the production of reactive oxygen/nitrogen species (ROS/RNS) (Cheignon et al., 2017). One of the proposed mechanisms through which Aβ triggers oxidative/nitrosative stress involves NMDA receptors (Kamat et al., 2016). The activation of NMDA receptors causes a huge Ca^2+^ influx into the postsynaptic neurons, followed by mitochondrial impairment, enhanced synthesis of nitric oxide, and excessive ROS/RNS production, which result in macromolecular damage and neurodegeneration (Milatovic et al., 2006).

Previous research showed that memantine treatment is cost-effective, safe, and connected with gains in quality-adjusted life-years, particularly for mild-to-moderate AD patients (Jiang and Jiang, 2015; Matsunaga et al., 2015; Tampi and van Dyck, 2007).

After oral administration, memantine is rapidly and fully absorbed, with an almost 100% bioavailability. The time to reach maximum plasma concentration (Cmax) following a single oral dose of 20 mg is 3–8 h, with a Cmax of 22–46 ng/ ml (Kornhuber et al., 2007). Absorption is not affected by food. Memantine displays linear pharmacokinetics in a wide range of doses, with a *t*_1/2_ between 60 and 70 h (Noetzli and Eap, 2013). Steady-state conditions are attained within 11 days. It has been reported that the volume of distribution (V_d_) is between 4 and 9 L/kg, and the plasma protein binding rate is approximately 45% (Kornhuber et al., 2007). Most of the memantine administered dose (75–90%) is expelled unchanged in urine, and its metabolites are inactive. Memantine is primarily excreted by the kidneys, with contributions of tubular secretion. It has been shown that memantine is a substrate of the organic cation transporter 2 (OCT2), which is mainly expressed in the kidneys and may be involved in memantine elimination (Ciarimboli, 2011). Urine pH is also a key factor for memantine renal excretion. In a clinical trial, it was demonstrated that renal clearance is seven- to ten-fold higher in alkaline urine (pH 8) than in acidic urine (pH 5) (Freudenthaler et al., 1998; Noetzli and Eap, 2013). Memantine acts as selective inhibitor of cytochrome P450 (CYP) 2B6 activity at clinically relevant dosages, without affecting other CYP enzymes (Kornhuber et al., 2007).

There have been a few clinical studies (cf. Rammes et al., 2008; Fonseca-Santos et al., 2015; Summerfield et al., 2016) that determined the penetration of memantine across the blood–brain barrier and confirmed its potential pharmacodynamic effects. The changes/fluctuations of memantine concentrations measured in human cerebrospinal fluid (CSF) between subsequent memantine doses have not been clearly described in the literature; clinical studies have only reported plasmatic levels, with subsequent pharmacokinetic profile calculations. At daily doses of 20 mg, the steady-state memantine plasma concentration ranges between 70 and 150 ng/ml (0.5–1 μM), with a great interpersonal variation (Parsons et al., 2007). One commonly accepted clinical study has determined that memantine can be detected in the CSF (Kornhuber and Quack, 1995). There are some clinical studies that have reported alterations in oxidative stress biomarkers in the CSF in patients with AD. Increased levels of oxidized/nitrated proteins (Tohgi et al., 1999; Aoyama et al., 2000; Ahmed et al., 2005), significant lipid peroxidation (Lovell et al., 1997; Montine et al., 1998; Praticò et al., 2000), oxidative damage to DNA (Lovell and Markesbery, 2001; Abe et al., 2002), and changes in antioxidant defense systems (Jimenez-Jimenez et al., 1997; Gumusyayla et al., 2016) were found in patients with AD when compared with healthy subjects. In the study of De Felice et al. (2007), Aβ oligomers (ADDL) dysregulated NMDA receptors function resulting in enhanced ROS formation in hippocampal neuronal cultures. It was observed that increased ROS formation was rather induced by direct activation of NMDA receptors by ADDLs than indirect overstimulation by glutamate released from ADDL-damaged neurons. More importantly, memantine completely protected ADDL-induced intraneuronal Ca^2+^ and ROS generation. Memantine also reduced glutamate-induced peroxynitrite generation mediated *via* NMDA receptors in brain-derived endothelial cells (Scott et al., 2007). Memantine was found also to be effective in the reduction of oxidative stress and prevented memory dysfunction in aged rats (Pietá Dias et al., 2007). In addition, memantine prevented oxidative stress induced by methylmercury (Liu et al., 2013) and carbofuran (Milatovic et al., 2005) in rats.

The purpose of this study is to explore the changes in the concentrations of memantine in the cerebrospinal fluid of AD patients, to evaluate its impact of the memantine administration over time, and to consider its pharmacodynamic effects by examining selected oxidative stress biomarkers in the CSF.

## Methods

### Sampling and Patients

We recruited 22 subjects at the Department of Neurology, University Hospital, Hradec Kralove, who were treated regularly with a standard therapeutic memantine dose (20 mg/day; standard routine—morning or evening administration) for at least 3 months before sample collection. Patients, all Caucasians, were divided into four groups depending on the time between memantine administration and plasma/CSF sampling: 6 ± 0.25 h (*n* = 5; 2 men and 3 women; aged 75.20 ± 1.51 years), 12 ± 0.25 h (*n* = 5; 3 men and 2 women; aged 70.50 ± 2.95 years), 18 ± 0.25 h (*n* = 6, 3 men and 3 women, aged 72.33 ± 3.09 years), and 24 ± 0.25 h (*n* = 6; 2 men and 4 women; aged 69.50 ± 2.68 years) following memantine administration. The time interval between whole blood and CSF sampling did not exceed 15 min. None of the patients in the group were taking urinary alkalizing drugs or had moderate-to-severe renal insufficiency, which could affect the pharmacokinetics of memantine (Noetzli and Eap, 2013).

An informed consent to lumbar puncture and to this clinical study was signed by all patients. This study was approved by the Ethics Committee of the University Hospital, Hradec Kralove (No. 201704 D01M). The project was also approved and supervised by the State Institute for Drug Control of Czech Republic (SUKL) and is registered in EU Clinical Trials Register (No. EudraCT 2016-004097-17), registered 06 October 2016, https://www.clinicaltrialsregister.eu/ctr-search/trial/2016-004097-17/CZ#P.

The diagnostic process was performed according to the national and international AD guidelines (Ressner et al., 2008; Hort et al., 2010), and all subjects met the National Institute of Neurological and Communication Disorders and Stroke and the Alzheimer’s Disease and Related Disorders Association (NINCDS-ADRDA) (McKhann et al., 2011) criteria for probable AD. All subjects received a magnetic resonance imaging (MRI), neuropsychological examination, laboratory examination, and lumbar puncture. The cerebrospinal fluid was withdrawn by a standard lumbar puncture, using a single-use traumatic needle. Dementia syndrome was established by clinical and neuropsychological examination by the American Psychiatric Association, according to the *Diagnostic and Statistical Manual of Mental Disorders*, 4th Edition (DSM-IV) criteria.

### Distribution Study

#### Reagents and Chemicals

Methanol, formic acid (both mass spectrometry gradient grade), memantine, and amantadine were purchased from Sigma-Aldrich (St. Louis, MO, USA). Ultrapure water was produced by Aqua Osmotic 06 (Aqua Osmotic, Tišnov, Czech Republic). Amantadine was used as an internal standard (IS). Supelco Supelclean LC-WCX (1 ml, 100 mg) solid-phase extraction (SPE) cartridges were purchased from Sigma-Aldrich (St. Louis, MO, USA).

#### Standard Solution and Calibration Standard

Stock solutions of memantine and amantadine (both 100 µg/ml) were prepared in ultrapure water. Working solutions at several concentrations were obtained by stock solution dilution. Calibration standards were prepared by spiking blank matrix (990 µl) with 10 µl of aliquots of working solution, to give concentrations in matrix of 2, 8, 20, 50, 100, 300, and 500 ng/ml. Stock solution was diluted to obtain the IS working solution (1 µg/ml).

#### Sample Preparation

CSF and plasma (both 1 ml) were spiked with 20 µl of IS working solution. After being spiked, the mixture was loaded onto an SPE Supelclean LC-WCX cartridge and pretreated with methanol (1 ml) and water (1 ml). The cartridge was washed with water (1 ml) and eluted with 5% formic acid in methanol (1 ml). The eluate was evaporated, using the rotational vacuum concentrators UNIVAPO 100 ECH (UniEquip GmbH, Germany). Residue was reconstituted with 500 µl of water. Finally, 5 µl of reconstituted samples was injected into the high-performance liquid chromatography–mass spectrometry (HPLC–MS) system.

#### LC–MS/MS Equipment and Parameters

LC–MS analysis was performed using a Finnigan Surveyor plus system (Thermo Scientific, San Jose, CA, USA), equipped with a quaternary MS pump with an integrated degasser and an autosampler with an integrated column oven. The chromatographic separation was performed on a Kinetex C18 column (5 µm, 50 mm × 4.6 mm ID) preceded by a C18 security guard cartridge (4.0 mm × 3.0 mm ID), both from Phenomenex (Torrance, CA, USA). Separation was attained using an isocratic elution with the flow rate of 0.5 ml/min. The column and tray temperature was set at 25°C and 10°C, respectively. The mobile phase consisted of 0.5% formic acid in water and methanol (45:55 v/v). The run time of the analysis was 3 min.

Mass spectrometry was performed on an LTQ XL linear ion trap instrument (Thermo Scientific, San Jose, CA, USA), coupled with heated electrospray ionization (HESI-II) probe operated in the positive ion mode. After optimization, the parameters in the source were set as follows: source voltage of 4.5 kV, source heater temperature of 250°C, sheath gas flow of 30 arb, and auxiliary gas flow of 10 arb. The mass spectrometer was operated in the selected reaction monitoring (SRM) channels. Fragment ion *m*/*z* 180 → 163 was used for memantine quantification, while *m*/*z* 152 → 135 was used for amantadine quantification. Thermo Fisher Xcalibur software was used for the analysis.

#### Method Validation

Seven-point calibration curves were generated using values ranging from 2 to 500 ng/ml of memantine in both plasma and CSF. The ratio of the memantine peak area to the IS peak area was plotted versus the concentration of memantine. The determination coefficients (*r* ^2^) were greater than 0.99 for both curves. The limit of quantification (LOQ) was defined as the lowest sample concentration measured with 20% accuracy and a relative error of ±20%. The LOQs for memantine were 2 ng/ml for both biological liquids (plasma and CSF).

Quality control samples were used to determine the extraction efficiency of memantine from human plasma and CSF. The recovery at three concentrations was determined by the comparison of peak areas obtained from the plasma sample with those of the standard solution spiked with blank plasma or with CSF residue. Recovery was stable for all concentrations of memantine, and recovery levels for plasma and CSF were 93.2% and 94.7% for 5 ng/ml, 95.1% and 94.2% for 50 ng/ml, and 92.8% and 93.6% for 250 ng/ml, respectively.

### Oxidative Stress Study

The method by Vanova et al. (2018) was used for the determination of oxidative stress biomarkers, malondialdehyde (MDA), and 3-nitrotyrosine (3-NT) and for thiol redox homeostasis evaluated by no-protein thiols (NP-SH) and non-protein disulfides (NP-SS-NP).

#### Determination of Malondialdehyde and 3-Nitrotyrosine Levels in Cerebrospinal Fluid

An aliquot of 50 μl of CSF was diluted with 200 μl of phosphate-buffered saline (PBS), mixed with 50 μl of 6 M of aqueous NaOH, and incubated at 60°C for 3 h in a metal block thermostat. Then, the sample was cooled on ice; and 50 μl of internal standard solution, 30 μM of glutaraldehyde (GLA), and 17 μM of stable-isotope-labeled 3-nitrotyrosine ([^13^C_9_]3-NT) were added. The mixture was precipitated by using 150 μl of 35% trichloroacetic acid (w/w) and centrifuged at 2,800×*g* at 4°C for 5 min. The supernatant obtained (450 μl) was derivatized with 50 μl of 5 mM of 2,4-dinitrophenylhydrazine solution [2% formic acid in acetonitrile (ACN), v/v] at 37°C for 1 h, avoiding direct light exposure and under constant shaking (300 RPM) using a thermo shaker incubator.

The derivatized sample was purified by SPE. A Phenomenex Strata® C18-E (55 μM, 70 Å) 100 mg/3 ml of SPE cartridge was equilibrated with MeOH (3 ml) and washed with water (3 ml). Then, 500 μl of sample was added to the cartridge and washed with 500 μl of buffer (10 mM of ammonium acetate + 0.1% acetic acid). Analytes were eluted with 1,000 μl of MeOH:buffer (96:4, v/v) solution. The eluent was dried in a vacuum concentrator centrifuge and reconstituted by adding 250 μl of buffer. The sample was then transferred into a vial and injected into the LC–MS/MS system.

#### Determination of Glutathione, Non-Protein Thiols, and Non-Protein Disulfides

For the determination of NP-SH, 100 μl of CSF was mixed with 1,000 μl of 0.15 M of PBS solution (pH 7.5) and 70 μl of DTNB solution (10 mg/ml, 0.15 M of PBS). After 10 min of reaction, samples were precipitated by adding 50 μL of 35% HCl and centrifuged at 6,800×*g* at 4°C for 5 min.

For the determination of NP-SS-NP, 100 μl of CSF was mixed with 200 μl of 0.5 M of PBS solution (pH 7.5) and 100 μl of sodium borohydride NaBH_4_ (100 mg/ml, 0.15 M of PBS); 50 μl of EtOH was added to reduce foaming. The mixture was incubated at 60°C for 30 min in an MBT-250 Metal Block Thermostat. Then, the sample was cooled on ice, and 40 μl of 18% HCl was added slowly to adjust the pH to 1 and remove residual NaBH_4_. Neutralization was achieved by addition of 610 μl of 0.5 M of PBS (pH 10). The samples were derivatized for 20 min with 70 μl of DTNB solution. The samples were precipitated by adding 50 μl of 35% HCl and centrifuged at 6,800×*g* at 4°C for 5 min.

Both supernatants were then transferred to vials and injected into the HPLC–UV system.

### Statistical Analysis

Statistical analysis was performed using IBM^®^ SPSS^®^ Statistics and Graph Pad Prism, version 5.0 (GraphPad Software, San Diego, California) software. Data were analyzed by non-parametric tests (Mann–Whitney *U* test, Kruskal–Wallis test). The level of significance was set at 2α = 0.05. All values are presented as the mean ± SEM (standard error of the mean).

## Results

In first part of study, the time-dependent fluctuations of the memantine concentration in plasma and CSF are summarized in **Table 1**. The plasma concentrations for all time intervals were relatively stable (approximately 105–111 ng/ml; computed without patient no. 10, as this patient was excluded for noncompliance); the plasma concentrations were higher after 24 h (216.01 ± 42.69 ng/ml), without reaching the statistical level of significance (Kruskal–Wallis test, *p* ≥ 0.2). The real memantine concentration in the CSF increased from 12 h (46.93 ± 13.94) to 24 h (91.75 ± 12.59), although the differences among the time intervals were not statistically significant (Kruskal–Wallis test, *p* ≥ 0.2). The ratio of plasma to CSF concentration (the results exclude those of patient nos. 9 and 10, as their CSF and/or plasma concentrations were under the limit of detection [LOD]) was also relatively stable, and all time intervals had an approximate value of 50% in most patients.

**Table 1:** Plasma and CSF memantine concentration overview.

Time (h)	Code	Age	Plasma (ng/ml)	CSF (ng/ml)	Ratio (%)
**6**	1	72	66.34	26.75	40.32
	2	71	80.38	20.53	25.54
	3	79	141.93	71.06	50.06
	4	79	126.96	62.71	49.39
	5	75	140.23	82.11	58.55
			**111.17 ± 14.18**	**52.63 ± 11.01**	**44.77 ± 5.03**
**12**	6	65	69.45	35.42	51.00
	7	73	126.96	80.50	63.41
	8	65	57.11	24.87	43.54
	9	79	175.07	0.00	0.00
	10	70	0.00	0.00	0.00
			**107.15 ± 23.63**	**46.93 ± 13.94**	**52.65 ± 4.74**
**18**	11	56	74.23	40.62	54.72
	12	78	180.72	144.39	79.9
	13	75	78.38	43.17	55.08
	14	76	27.84	14.05	50.47
	15	72	78.95	32.77	41.51
	16	77	190.77	99.08	51.94
			**105.15 ± 24.46**	**62.35 ± 10.41**	**55.60 ± 4.82**
**24**	17	71	326.39	146.97	45.03
	18	75	131.11	79.92	60.96
	19	57	334.94	54.62	16.31
	20	68	114.21	76.63	67.1
	21	72	173.42	117.10	67.53
	22	74	118.36	75.24	63.57
			**199.74 ± 38.60**	**91.75 ± 12.59**	**53.42 ± 7.44**

Bold texts: Group results are the mean ± SEM (the zero values excluded).

The overview of oxidative stress markers is summarized in **Table 2**. Significant differences in the levels of 3-NT, NP-SH, and NP-SS-NP between the group treated with memantine and the control group (Mann–Whitney *U* test, all *p* ≤ 0.05) were observed. 3-NT levels were lower in patients receiving treatment (−18%), indicating that memantine might have a protective role in protein nitration. Levels of both non-protein thiols and disulfides were increased in the treated group as compared with the control group (+55 and +10%, Mann–Whitney *U* test, all *p* < 0.05). No significant differences were observed in the levels of MDA or glutathione (GSH) (Mann–Whitney *U* test, all *p* > 0.05).

**Table 2:** CSF concentration of markers in patients treated with memantine versus the control group.

Marker	3-NT	MDA	GSH	NP-SH	NP-SS-NP
	nmol/ml	nmol/ml	nmol/ml	µg/ml	µg/ml
Control AD patients	0.092 ± 0.037	9.043 ± 3.414	0.354 ± 0.040	0.986 ± 0.099	3.011 ± 4.469
AD patients treated with memantine	0.075 ± 0.026	9.349 ± 2.020	0.426 ± 0.192	1,529 ± 0.543	3.326 ± 1.434
	*			*	*

*Indicates a statistically significant difference (Mann–Whitney U test, p ≤ 0.05). Concentrations of 3-NT, MDA, and GSH are in nmol/ml; concentrations of NP-SH and NP-SS-NP are in μg/ml. The mean CSF level of memantine (n = 22) in the treated group was 65.33 ± 9.4 ng/ml.

## Discussion

Memantine, the NMDA receptor antagonist, is clinically tolerated and widely used in AD treatment. Memantine has relatively few side effects in the therapeutic range (Parsons et al., 1999) and has a potentially promising pharmacokinetic profile. Although it is known that memantine is a cationic amphiphilic substance and could readily pass through biological membranes in its unionized form by passive diffusion (Honegger et al., 1993), clinical studies describing the blood–brain barrier permeation or the targeting of brain structures are really rare. In our study, the memantine concentrations were measured in the plasma and in the CSF. According to previously published study, the CSF concentration may closely reflect the concentration in brain tissues, which is freely available to interact with NMDA receptors, due to memantine lysosomal accumulation (Kornhuber and Quack, 1995).

This is the second study examining the memantine concentration in the CSF. The previously published study by Kornhuber and Quack (1995) determined a high correlation between serum and CSF concentrations in humans, with a mean ratio of 0.52 ± 0.094 (*n* = 5). This clear conclusion may be questioned by the setting of this study, as only six patients were enrolled and only three of them ever theoretically achieved a steady state (administration of memantine for at least 11 days). The dose range was 5–30 mg/day, and in one patient, memantine was administered intravenously. This small clinical study is the only source that is cited in the human pharmacokinetics section in the freely available materials from Forest Laboratories, Inc. In Food and Drug Administration (FDA)-approved materials, there is only one clinical study that describes CSF bioavaibility (FDA, 2003), but the results were revised, and the plasma/brain ratio information is not available. The CSF concentrations correspond with our findings following an i.v. infusion of 20-mg dose of memantine (*n* = 5; patients with Parkinson’s disease and patients with involuntary movements and pain symptoms included). This study just indicates that memantine was detected in the CSF within 26 min after the infusion (FDA, 2003).

Conversely, in our larger cohort (*n* = 22; patients with AD), a single dose of memantine was 20 mg/day in tablet form, and patients were treated for at least 3 months. Other factors that may affect memantine pharmacokinetics include sex (higher plasma concentrations in women), genetics (Noetzli et al., 2013), and a reduction in the clearance of memantine with increased age; aging is also associated with lower body weight and the concurrent administration of drugs secreted by tubular secretion (Kornhuber et al., 2007). All of these factors contribute to the high inter-individual variability in the pharmacokinetics of memantine.

We found that CSF and plasma concentrations fluctuate considerably in relation to the elapsed time from administration. The plasma/CSF ratios at all time intervals were within the range of 45.89% (6 h after application) to 55.60% (18 h after administration), which corresponds with previously published findings (Kornhuber et al., 2007). However, three patients had CSF/plasma ratios that were significantly lower; patient nos. 2, 9, and 19 had ratios in the 0.00–25.54% range. Despite inter-individual differences, it is assumed that both the plasma and CSF levels of memantine are more stable among individual patients than are those of donepezil (Vališ et al., 2017).

The other aim of our study was to deduce whether the brain concentration would be sufficient to block NMDA receptors. According to previously published data, the IC_50_ value of memantine as an NMDA antagonist is in the micromolar range, from 0.52 µM (GluN2C) to 0.80 µM (GluN2A) (Traynelis et al., 2010). These values correspond to a range of 112–173 ng/ml. The majority of the CSF levels reported in our study fall below the lower limit; the memantine concentration exceeded the upper range in three CSF samples. However, according to Kornhuber and Quack, (1995), the submicromolar range of memantine may specifically interact with the NMDA receptor PCP binding site. Based on our results, the memantine CSF concentration was lower than 25% of the IC_50_ value in five cases (patient no. 10 was excluded for noncompliance), and this corresponds with one quarter of the patients enrolled in this study.

The other possibility for demonstrating the efficacy of some drugs is to evaluate their pharmacodynamic effects. It has been demonstrated that the neuronal damage induced by oxidative stress is associated with the alteration of NMDA receptor activity. Although, Ca^2+^ overload through synaptic NMDA receptors is not regarded as being neurotoxic, the overstimulation of extra-synaptic NMDA receptors (located on dendrites or the sides of spines) triggers excitotoxicity. In therapeutic doses, memantine is reported to preferentially block extra-synaptic NMDA receptors over synaptic NMDA receptors (Wang et al., 2013; Zhang et al., 2016). In the study by De Felice et al. (2007), Aβ oligomers (ADDL) dysregulated NMDA receptor function, resulting in enhanced ROS formation in hippocampal neuronal cultures. It was observed that increased ROS formation was induced by the direct activation of NMDA receptors by ADDLs rather than by the indirect overstimulation from glutamate released by ADDL-damaged neurons. More importantly, memantine completely protected against ADDL-induced intra-neuronal Ca^2+^ and ROS generation. Memantine also reduced glutamate-induced peroxynitrite generation mediated by NMDA receptors in brain-derived endothelial cells (Scott et al., 2007). It was also found that memantine reduces oxidative stress and prevents memory dysfunction in aged rats (Pietá Dias et al., 2007). In addition, memantine prevented oxidative stress induced by methylmercury (Liu et al., 2013) and carbofuran (Milatovic et al., 2005) in rats.

In this study, we compared alterations in oxidative stress parameters in the CSF of patients treated with memantine compared with the untreated control group. A decline in the antioxidant defense system is the initial step of oxidative stress. Thiol–disulfide homeostasis reflects the ability of an organism to maintain the redox balance (Gumusyayla et al., 2016). Therapy with memantine positively affected the levels of thiols, but the formation of disulfides was also increased.

The levels of 3-NT (a marker of protein nitration) in the CSF of AD patients vary from negligible to significant, as compared with that of age-matched control subjects (Tohgi et al., 1999; Ahmed et al., 2005; Ryberg and Caidahl, 2007; Korolainen and Pirttila, 2009). The activation of NMDA receptors generates nitric oxide and superoxide, which cause the formation of peroxynitrite, a potent nitrating and oxidizing agent (Scott et al., 2007). In our study, memantine associated with decreasing of 3-NT levels, suggesting its ameliorative effect on protein nitration. However, levels of the lipid peroxidation product MDA were not significantly altered. Generally, lipid peroxidation products (e.g., isoprostanes, 4-hydroxy-2-trans nonenal, and acrolein) were elevated in the CSF of AD patients (Butterfield et al., 2001; Arlt et al., 2002; Pratico et al., 2002; Montine et al., 2004). Unfortunately, no specific data for MDA levels in the CSF of AD patients are available.

This study raises the issue further and provides data on the concentration of memantine in the CSF and possible proof of memantine efficacy through the evaluation of oxidative stress parameters. Unfortunately, a significant correlation between changes in oxidative stress parameters and an appropriate memantine CSF concentration was not found. One limitation of our study was the difficulty in recruiting patients consenting to undergo a lumbar puncture. Thus, the sample size was relatively small.

Moreover, more data need to be measured/evaluated to find out some relationship to current treatment options, including other symptomatic therapies, such as rivastigmine, donepezil, and galantamine. The presented memantine data complement our previous donepezil study, which revealed larger heterogeneity. The findings of this study can have impact on clinical practice, including dosing schemes and the role of compliance in AD patients.

## Conclusions

The results of this study indicate that the changes in memantine concentration in the cerebrospinal fluid of AD patients in relation to time of administration are negligible. The ratios of the CSF and plasma memantine concentrations over time were relatively stable in most patients. However, the memantine CSF concentration was lower than 25% of IC_50_ value (as an NMDA antagonist) in approximately one quarter of cases of the patients enrolled in this study. However, it appears that memantine therapy positively affected the levels of some oxidative stress parameters, especially thiols and 3-NT. Based on our results, it can be assumed that the memantine therapeutic effect of up to one quarter of patients is limited.

## Data Availability

All datasets generated for this study are included in the manuscript.

## Ethics Statement

The studies involving human participants were reviewed and approved by Ethics Committee of the University Hospital, Hradec Kralove (No. 201704 D01M). The patients/participants provided their written informed consent to participate in this study.

## Author Contributions

MV, JaM, OV, and ZP performed the blood and CSF sampling and data collection. JZK and MV designed study. DH performed the HPLC analysis. NV performed the oxidative stress study. JiM analyzed data. MV, NV, and JZK wrote manuscript. BK, JH, and KK commented and revised the text of the manuscript. All authors read and approved the final manuscript.

## Funding

This study was supported by the project IT4Neuro(degeneration), reg. nr. CZ.02.1.01/0.0/0.0/18_069/0010054.

## Conflict of Interest Statement

The authors declare that the research was conducted in the absence of any commercial or financial relationships that could be construed as a potential conflict of interest.

## Abbreviations

3-NT, 3-nitrotyrosine; ADDL, Aβ oligomers; AD, Alzheimer’s disease; AChE, acetylcholinesterase; AUCss, area under the concentration–time curve at steady state; BBB, blood–brain barrier; Cap, capsule; Cmax,ss, maximum steady-state plasma drug concentration during a dosage interval; Cltot, total systemic clearance; CSF, cerebrospinal fluid; CYP, cytochrome P450; CV, coefficient of variation; ER, extended-release formulation; FDA, Food and Drug Administration; GSH, glutathione (GSH) and non-protein thiols (NP-SH); HPLC, high-performance liquid chromatography; IR, immediate-release formulation; LOD, limit of detection; MDA, malondialdehyde; NMDA, *N*-methyl-d-aspartate; NP-SH, non-protein thiols; NP-SS-NP, non-protein disulfides; P-gp, P-glycoprotein; ROS/RNS, oxygen/nitrogen species; SEM, standard error of the mean; SR, sustained-release formulation; *t*_1/2_, elimination half-life; tmax,ss, time to reach Cmax,ss; UGT, uridine 50-diphosphoglucuronosyltransferase; V_d_, apparent volume of distribution

## References

[B1] Abe T. Tohgi H. Isobe C. Murata T. Sato C. (2002). Remarkable increase in the concentration of 8-hydroxyguanosine in cerebrospinal fluid from patients with Alzheimer’s disease. J. Neurosci. Res. 70, 447–450. 10.1002/jnr.10349 12391605

[B2] Ahmed N. Ahmed U. Thornalley P. J. Hager K. Fleischer G. Munch G. (2005). Protein glycation, oxidation and nitration adduct residues and free adducts of cerebrospinal fluid in Alzheimer’s disease and link to cognitive impairment. J. Neurochem. 92, 255–263. 10.1111/j.1471-4159.2004.02864.x 15663474

[B3] Aoyama K. Matsubara K. Fujikawa Y. Nagahiro Y. Shimizu K. Umegae N. (2000). Nitration of manganese superoxide dismutase in cerebrospinal fluids is a marker for peroxynitrite-mediated oxidative stress in neurodegenerative diseases. Ann. Neurol. 47, 524–527. 10.1002/1531-8249(200004)47:4<524::AID-ANA19>3.0.CO;2-5 10762167

[B4] Arlt S. Beisiegel U. Kontush A. (2002). Lipid peroxidation in neurodegeneration: new insights into Alzheimer’s disease. Curr. Opin. Lipidol. 13, 289–294. 10.1097/00041433-200206000-00009 12045399

[B5] Butterfield D. A. Drake J. Pocernich C. Castegna A. (2001). Evidence of oxidative damage in Alzheimer’s disease brain: central role for amyloid beta-peptide. Trends Mol. Med. 7, 548–554. 10.1016/S1471-4914(01)02173-6 11733217

[B6] Cheignon C. Tomas M. Bonnefont-Rousselot D. Faller P. Hureau C. Collin F. (2017). Oxidative stress and the amyloid beta peptide in Alzheimer’s disease. Redox. Biol. 14, 450–464. 10.1016/j.redox.2017.10.014 29080524 PMC5680523

[B7] Ciarimboli G. (2011). Role of organic cation transporters in drug-induced toxicity. Expert Opin. Drug Metab. Toxicol. 7, 159–174. 10.1517/17425255.2011.547474 21241199

[B8] De Felice F. G. Velasco P. T. Lambert M. P. Viola K. Fernandez S. J. Ferreira S. T. (2007). Abeta oligomers induce neuronal oxidative stress through an *N*-methyl-D-aspartate receptor-dependent mechanism that is blocked by the Alzheimer drug memantine. J. Biol. Chem. 282, 11590–11601. 10.1074/jbc.M607483200 17308309

[B9] FDA (2003). Available from: https://www.accessdata.fda.gov/drugsatfda_docs/nda/2003/21-487_Namenda_Bioeqr_P2.pdf (Accessed October 16, 2003).

[B10] Fonseca-Santos B. Gremiao M. P. D. Chorilli M. (2015). Nanotechnology-based drug delivery systems for the treatment of Alzheimer’s disease. Int. J. Nanomedicine. 10, 4981–5003. 10.2147/IJN.S87148 26345528 PMC4531021

[B11] Freudenthaler S. Meineke I. Schreeb K. H. Boakye E. Gundert-Remy U. Gleiter C. H. (1998). Influence of urine pH and urinary flow on the renal excretion of memantine. Br. J. Clin. Pharmacol. 46, 541–546. 10.1046/j.1365-2125.1998.00819.x 9862242 PMC1873797

[B12] Gumusyayla S. Vural G. Bektas H. Deniz O. Neselioglu S. Erel O. (2016). A novel oxidative stress marker in patients with Alzheimer’s disease: dynamic thiol–disulphide homeostasis. Acta. Neuropsychiatry 28, 315–320. 10.1017/neu.2016.13 27040443

[B13] Honegger U. E. Quack G. Wiesmann U. N. (1993). Evidence for lysosomotropism of memantine in cultured human cells: cellular kinetics and effects of memantine on phospholipid content and composition, membrane fluidity and beta-adrenergic transmission. Pharmacol. Toxicol. 73, 202–208. 10.1111/j.1600-0773.1993.tb01564.x 8295847

[B14] Hort J. O’Brien J. T. Gainotti G. Pirttila T. Popescu B. O. Rektorova I. (2010). EFNS guidelines for the diagnosis and management of Alzheimer’s disease. Eur. J. Neurol. 17, 1236–1248. 10.1111/j.1468-1331.2010.03040.x 20831773

[B15] Huang W. J. Zhang X. Chen W. W. (2016). Role of oxidative stress in Alzheimer’s disease. Biomed. Rep. 4, 519–522. 10.3892/br.2016.630 27123241 PMC4840676

[B16] Jiang J. Jiang H. (2015). Efficacy and adverse effects of memantine treatment for Alzheimer’s disease from randomized controlled trials. Neurol. Sci. 36, 1633–1641. 10.1007/s10072-015-2221-2 25899425

[B17] Jimenez-Jimenez F. J. de Bustos F. Molina J. A. Benito-Leon J. Tallon-Barranco A. Gasalla T. (1997). Cerebrospinal fluid levels of alpha-tocopherol (vitamin E) in Alzheimer’s disease. J. Neural Transm. 104, 703–710. 10.1007/BF01291887 9444569

[B18] Kamat P. K. Kalani A. Rai S. Swarnkar S. Tota S. Nath C. (2016). Mechanism of oxidative stress and synapse dysfunction in the pathogenesis of Alzheimer’s disease: understanding the therapeutics strategies. Mol. Neurobiol. 53, 648–661. 10.1007/s12035-014-9053-6 25511446 PMC4470891

[B19] Klimova B. Maresova P. Kuca K. (2016). Non-pharmacological approaches to the prevention and treatment of Alzheimer’s disease with respect to the rising treatment costs. Curr. Alzheimer Res. 13, 1249–1258. 10.2174/1567205013666151116142302 26567732

[B20] Klimova B. Valis M. Kuca K. (2017). Cognitive decline in normal aging and its prevention: a review on non-pharmacological lifestyle strategies. Clin, Interv. Aging 12, 903–910. 10.2147/CIA.S132963 28579767 PMC5448694

[B21] Kornhuber J. Kennepohl E. M. Bleich S. Wiltfang J. Kraus T. Reulbach U. (2007). Memantine pharmacotherapy. Clin. Pharmacokinet. 46, 599–612. 10.2165/00003088-200746070-00005 17596105

[B22] Kornhuber J. Quack G. (1995). Cerebrospinal fluid and serum concentrations of the *N*-methyl-D-aspartate (NMDA) receptor antagonist memantine in man. Neurosci. Lett. 195, 137–139. 10.1016/0304-3940(95)11785-U 7478269

[B23] Korolainen M. A. Pirttila T. (2009). Cerebrospinal fluid, serum and plasma protein oxidation in Alzheimer’s disease. Acta. Neurol. Scand. 119, 32–38. 10.1111/j.1600-0404.2008.01057.x 18547271

[B24] Liu W. Xu Z. Deng Y. Xu B. Wei Y. Yang T. (2013). Protective effects of memantine against methylmercury-induced glutamate dyshomeostasis and oxidative stress in rat cerebral cortex. Neurotox. Res. 24, 320–337. 10.1007/s12640-013-9386-3 23504438

[B25] Lovell M. A. Ehmann W. D. Mattson M. P. Markesbery W. R. (1997). Elevated 4-hydroxynonenal in ventricular fluid in Alzheimer’s disease. Neurobiol. Aging 1997, 18:457–18:461. 10.1016/S0197-4580(97)00108-5 9390770

[B26] Lovell M. A. Markesbery W. R. (2001). Ratio of 8-hydroxyguanine in intact DNA to free 8-hydroxyguanine is increased in Alzheimer disease ventricular cerebrospinal fluid. Arch. Neurol. 58, 392–396. 10.1001/archneur.58.3.392 11255442

[B27] Matsunaga S. Kishi T. Nakao Iwata N. (2015). Memantine monotherapy for Alzheimer’s disease: a systematic review and meta-analysis. PLoS One 10, 1–16. 10.1371/journal.pone.0123289 PMC4393306 25860130

[B28] McKhann G. Knopman D. S. Chertkow H. Hyman B. T. Jack C.R. Kawas C. H. (2011). The diagnosis of dementia due to Alzheimer’s disease: recommendations from the National Institute on Aging-Alzheimer’s Association workgroups on diagnostic guidelines for Alzheimer’s disease. Alzheimers Dement. 7 (3), 263–269. 10.1016/j.jalz.2011.03.005 21514250 PMC3312024

[B29] Milatovic D. Gupta R. C. Aschner M. (2006). Anticholinesterase toxicity and oxidative stress. Sci. World J. 6, 295–310. 10.1100/tsw.2006.38 PMC5917369 16518518

[B30] Milatovic D. Gupta R. C. Dekundy A. Montine T. J. Dettbarn W. D. (2005). Carbofuran-induced oxidative stress in slow and fast skeletal muscles: prevention by memantine and atropine. Toxicology. 208 (1), 13–24. 10.1016/j.tox.2004.11.004 15664429

[B31] Montine T. J. Markesbery W. R. Morrow J. D. Roberts L. J. 2nd (1998). Cerebrospinal fluid F2-isoprostane levels are increased in Alzheimer’s disease. Ann. Neurol. 44, 410–413. 10.1002/ana.410440322 9749613

[B32] Montine K. S. Quinn J. F. Zhan J. Fessel J. P. RobertsII L. J. Morrow J. D. (2004). Isoprostanes and related products of lipid peroxidation in neurodegenerative diseases. Chem. Phys. Lipids 128, 117–124. 10.1016/j.chemphyslip.2003.10.010 15037157

[B33] Noetzli M. Eap C. B. (2013). Pharmacodynamic, pharmacokinetic and pharmacogenetic aspects of drugs used in the treatment of Alzheimer’s disease. Clin, Pharmacokinet. 52, 225–241. 10.1007/s40262-013-0038-9 23408070

[B34] Noetzli M. Guidi M. Ebbing K. Eyer S. Wilhelm L. Michon A. (2013). Population pharmacokinetic study of memantine: effects of clinical and genetic factors. Clin. Pharmacokinet. 52, 211–223. 10.1007/s40262-013-0032-2 23371894

[B35] Parsons C. G. Danysz W. Quack G. (1999). Memantine is a clinically well tolerated *N*-methyl-D-aspartate (NMDA) receptor antagonist—a review of preclinical data. Neuropharmacology 38, 735–767. 10.1016/S0028-3908(99)00019-2 10465680

[B36] Parsons C. G. Stoffler A. Danysz W. (2007). Memantine: a NMDA receptor antagonist that improves memory by restoration of homeostasis in the glutamatergic system—too little activation is bad, too much is even worse. Neuropharmacology 53, 699–723. 10.1016/j.neuropharm.2007.07.013 17904591

[B37] Pietá Dias C. Martins de Lima M. N. Presti-Torres J. Dornelles A. Garcia V. A. Siciliani Scalco F. (2007). Memantine reduces oxidative damage and enhances long-term recognition memory in aged rats. Neuroscience 146 (4), 1719–1725. 10.1016/j.neuroscience.2007.03.018 17445991

[B38] Praticò D. Clark C. M. Lee V. M. Y. Trojanowski J. Q. Rokach J. FitzGerald G. A. (2000). Increased 8,12-*iso*-iPF_2α_-VI in Alzheimer’s disease: correlation of a noninvasive index of lipid peroxidation with disease severity. Ann. Neurol. 48, 809–812. 10.1002/1531-8249(200011)48:5<809::AID-ANA19>3.0.CO;2-9 11079549

[B39] Pratico D. Clark C. M. Liun F. Rokach J. Lee V. Y. Trojanowski J. Q. (2002). Increase of brain oxidative stress in mild cognitive impairment: a possible predictor of Alzheimer disease. Arch. Neurol. 59, 972–976. 10.1001/archneur.59.6.972 12056933

[B40] Rammes G. Danysz W. Parsons C. G. (2008). Pharmacodynamics of memantine: an update. Curr. Neuropharmacol. 6 (1), 55–78. 10.2174/157015908783769671 19305788 PMC2645549

[B41] Ressner P. Hort J. Rektorová I. Bartoš A. Rusina R. Línek V. (2008) Doporučené postupy pro diagnostiku Alzheimerovy nemoci a dalších onemocnění spojených s demencí. Cesk Slov Neurol N. 71/104 (4), 494–501.

[B42] Ryberg H. Caidahl K. (2007). Chromatographic and mass spectrometric methods for quantitative determination of 3-nitrotyrosine in biological samples and their application to human samples. J. Chromatogr. B. Analyt. Technol. Biomed. Life Sci. 851, 160–171. 10.1016/j.jchromb.2007.02.001 17344105

[B43] Scott G. S. Bowman S. R. Smith T. Flower R. J. Bolton C. (2007). Glutamate-stimulated peroxynitrite production in a brain-derived endothelial cell line is dependent on *N*-methyl-D-aspartate (NMDA) receptor activation. Biochem. Pharmacol. 73, 228–236. 10.1016/j.bcp.2006.09.021 17118345 PMC1855445

[B44] Summerfield S. G. Zhang Y. Liu H. (2016). Examining the uptake of central nervous system drugs and candidates across the blood–brain barrier. J. Pharmacol. Exp. Ther. 358 (2), 294–305. 10.1124/jpet.116.232447 27194478

[B45] Tampi R. R. van Dyck C. H. (2007). Memantine: efficacy and safety in mild-to-severe Alzheimer’s disease. Neuropsychiatry Dis. Treat, 3:245–258. 10.2147/nedt.2007.3.2.245 PMC2654628 19300557

[B46] Tohgi H. Abe T. Yamazaki K. Murata T. Ishizaki E. Isobe C. (1999). Alterations of 3-nitrotyrosine concentration in the cerebrospinal fluid during aging and in patients with Alzheimer’s disease. Neurosci. Lett. 269, 52–54. 10.1016/S0304-3940(99)00406-1 10821643

[B47] Traynelis S. F. Wollmuth L. P. McBain C. J. Menniti F. S. Vance K. M. Ogden K. K. (2010). Glutamate receptor ion channels: structure, regulation, and function. Pharmacol. Rev. 62, 405–496. 10.1124/pr.109.002451 20716669 PMC2964903

[B48] ValiŠ M. Masopust J. Vysata O. Hort J. Dolezal R. Tomek J. (2017). Concentration of donepezil in the cerebrospinal fluid of AD patients: evaluation of dosage sufficiency in standard treatment strategy. Neurotox. Res. 31, 162–168. 10.1007/s12640-016-9672-y 27718143 PMC5209410

[B49] Vanova N. Muckova L. Schmidt M. Herman D. Dlabkova A. Pejchal J. (2018). Simultaneous determination of malondialdehyde and 3-nitrotyrosine in cultured human hepatoma cells by liquid chromatography-mass spectrometry. Biomed. Chromatogr. 32 (12), e4349, 10.1002/bmc.4349 30051494

[B50] Wang Z. C. Zhao J. Li S. (2013). Dysregulation of synaptic and extrasynaptic N-methyl-D-aspartate receptors induced by amyloid-beta. Neurosci. Bull. 29, 752–760. 10.1007/s12264-013-1383-2 24136243 PMC5562550

[B51] Zhang Y. Li P. Feng J. Wu M. (2016). Dysfunction of NMDA receptors in Alzheimer’s disease. Neurol. Sci. 37, 1039–1047. 10.1007/s10072-016-2546-5 26971324 PMC4917574

[B52] Zhao Y. Zhao B. (2013). Oxidative stress and the pathogenesis of Alzheimer’s disease. Oxid. Med. Cell. Longev. 2013, 316523 10.1155/2013/316523. 23983897 PMC3745981

